# The Treatment of Rhodiola Mimics Exercise to Resist High-Fat Diet-Induced Muscle Dysfunction via Sirtuin1-Dependent Mechanisms

**DOI:** 10.3389/fphar.2021.646489

**Published:** 2021-04-15

**Authors:** Baiyang You, Yaoshan Dun, Siqian Fu, Dake Qi, Wenliang Zhang, Yuan Liu, Ling Qiu, Murong Xie, Suixin Liu

**Affiliations:** ^1^ Division of Cardiac Rehabilitation, Department of Physical Medicine and Rehabilitation, Xiangya Hospital of Central South University, Changsha, China; ^2^ National Clinical Research Center for Geriatric Disorders, Xiangya Hospital of Central South University, Changsha, China; ^3^ Division of Preventive Cardiology, Department of Cardiovascular Medicine, Mayo Clinic, Rochester, MN, United States; ^4^ College of Pharmacy, University of Manitoba, Winnipeg, MB, Canada

**Keywords:** salidroside, SIRT1, atrogenes, mitochondrion, rhodiola

## Abstract

Muscle dysfunction is a complication of high-fat diet (HFD)-induced obesity that could be prevented by exercise, but patients did not get enough therapeutic efficacy from exercise due to multiple reasons. To explore alternative or supplementary approaches to prevent or treat muscle dysfunction in individuals with obesity, we investigated the effects of Rhodiola on muscle dysfunction as exercise pills. SIRT1 might suppress atrogenes expression and improve mitochondrial quality control, which could be a therapeutic target stimulated by exercise and Rhodiola, but further mechanisms remain unclear. We verified the lipid metabolism disorders and skeletal muscle dysfunction in HFD feeding mice. Moreover, exercise and Rhodiola were used to intervene mice with a HFD. Our results showed that exercise and Rhodiola prevented muscle atrophy and dysfunction in obese mice and activating the SIRT1 pathway, while atrogenes were suppressed and mitochondrial quality control was improved. EX-527, SIRT1 inhibitor, was used to validate the essential role of SIRT1 in salidroside benefit. Results of cell culture experiment showed that salidroside alleviated high palmitate-induced atrophy and mitochondrial quality control impairments, but these improvements of salidroside were inhibited by EX-527 in C2C12 myotubes. Overall, Rhodiola mimics exercise that activates SIRT1 signaling leading to improvement of HFD-induced muscle dysfunction.

## Introduction

Skeletal muscle dysfunction (or muscle wasting) characterized with a reduction of myofibrillar, and mitochondrial dysfunction has been reported as an important complication of high-fat diet (HFD)-induced obesity (Perry et al., 2016). However, compared to its adverse impacts on type 2 diabetes, cardiovascular, or osteoarticular diseases, muscle dysfunction has received less attention. Thus, investigating the mechanisms and potential therapy against HFD-induced muscle dysfunction will have important clinical significance. Though the underlying mechanisms are not thoroughly understood, several factors are well known, for example, the oversupplied energy and impaired lipid metabolism damage the mitochondrial which is essential in maintain muscle quality (Jorgensen et al., 2015)**.**

A major therapeutic strategy against muscle dysfunction is exercise that effectively increases muscle mass, strength, and physical performance (Pillard et al., 2011; Dun et al., 2019a; Dun et al., 2019b). Although the molecular mechanisms of exercise in regulating HFD-induced muscle dysfunction are largely unknown, previous evidence indicated that exercise could decrease transcription of atrogenes, such as muscle-specific RING finger protein 1 (MuRF1) and atrogin1, both of which inhibit protein degradation and facilitate myofibrillar synthesis. Exercise also promotes mitochondrial mass and functions (Sandri et al., 2006). Mitochondrial quality control (MQC), including mitochondrial biogenesis, mitophagy, and fusion/fission, has been used as an important evaluation for mitochondrial function. Our previous studies indicated that exercise activates MQC in skeletal and myocardial muscle (Xie et al., 2019; Jiang et al., 2021). However, exercise lacks a long-term therapeutic efficacy for muscle dysfunction due to poor compliance or disabilities resulted from skeleton-muscle system (Conraads et al., 2012). Therefore, alternative or supplementary approaches to prevent muscle dysfunction in individuals with overweight need to be explored.

Sirtuin 1 (SIRT1), a sirtuin family protein, displays a nicotinamide adenine dinucleotide (NAD) protein deacetylase activity (Milne and Denu, 2008). Thus, it can be activated by changes of NAD+ levels during exercise. SIRT1 is associated with beneficial effects of exercise through resisting protein degradation and improving the quality of mitochondria (Tonkin et al., 2012). Overexpression of SIRT1 reduces muscle dysfunction by inhibiting key atrogenes and activating peroxisome proliferator-activated receptor gamma coactivator 1-α (PGC-1α) in skeletal muscle (Lee and Goldberg, 2013). SIRT1 also modulates transcription factors, such as FOXO3/PINK1/Parkin and MFN1/DRP1 that participate in the regulation of mitophagy and mitochondrial dynamics (Valero, 2014; Lei et al., 2020). Thus, SIRT1 might be a key therapeutic target for muscle atrophy or dysfunction stimulated by exercise.

*Rhodiola* is a natural plant which has been widely used to treat altitude sickness for hundreds of years (Kelly, 2001). Salidroside, an active ingredient from *Rhodiola* roots, activates the SIRT1 pathway to ameliorate diabetic nephropathy and hypoxia-induced neurodegeneration in mice via promoting mitochondrial biogenesis (Barhwal et al., 2015; Xue et al., 2019). More recently, salidroside was also found to improve denervation-induced muscle proteolysis and muscle atrophy (Wu et al., 2019). Our previous study also indicated that *Rhodiola chrysanthemifolia subsp. sacra (Raym.-Hamet) H.Ohba* (*R. sacra*) supplementary and exercise activated MQC of skeletal muscle, which contributes to the enhancement of exercise capacity and facilitates the muscle ability to resist fatigue in healthy mice (Dun et al., 2017). However, the effects of *R. sacra* on HFD-induced mitochondrial dysfunction and muscle atrophy remain unknown. Thus, in the present study, we explored the role of SIRT1 signaling pathway in mediating muscle dysfunction and compared the potential therapeutic effects of *R. sacra* and exercise.

## Materials and Methods

### Animals

Male C57BL/6J mice (8 weeks old) were purchased from the Laboratory Animal Centre, Xiangya Medical School (Changsha, Hunan, China). The mice were housed in temperature-controlled (22°C ± 2°C) quarters with a 12:12 hour light-dark cycle and free access to water and food. All animal procedures were in accordance with the guidelines for the use of live animals of National Institute of Health and were approved by the Medicine Animal Welfare Committee of Xiangya Medical School, Central South University (Changsha, China) (approval ID: SYXK 2015-0017).

### Groups

After one-week feeding adaptation, mice were randomly divided into four groups (*n* = 16 for each): normal chow (NC), high fat diet (HFD), HFD + *R. sacra* (HFD + RS) and HFD + exercise (HFD+EX). The HFD contains 45% of fat. The mice in the HFD + EX group were trained for exercise as described previously (Dun et al., 2017). In the HFD+RS group, mice were given 50 mg/ml of *R. sacra* per 500 mg/kg weight (Tibet Rhodiola Pharmaceutical Holding Company, Tibet, China) by gavage (0.1 ml/10 g weight) daily between 9 to 10 a.m. as previously described (Dun et al., 2017). The rest groups obtained normal saline by gavage at a dose of 0.1 ml per 10 g weight. Exercise, *R. sacra* and normal saline were administered together with HFD for 8 consecutive weeks. Body weights were weekly monitored during the whole experiment. An additional *R. sacra* treatment to the HFD mice didn’t cause any observable change in food intake pattern or gastrointestinal reaction.

**FIGURE 1:**
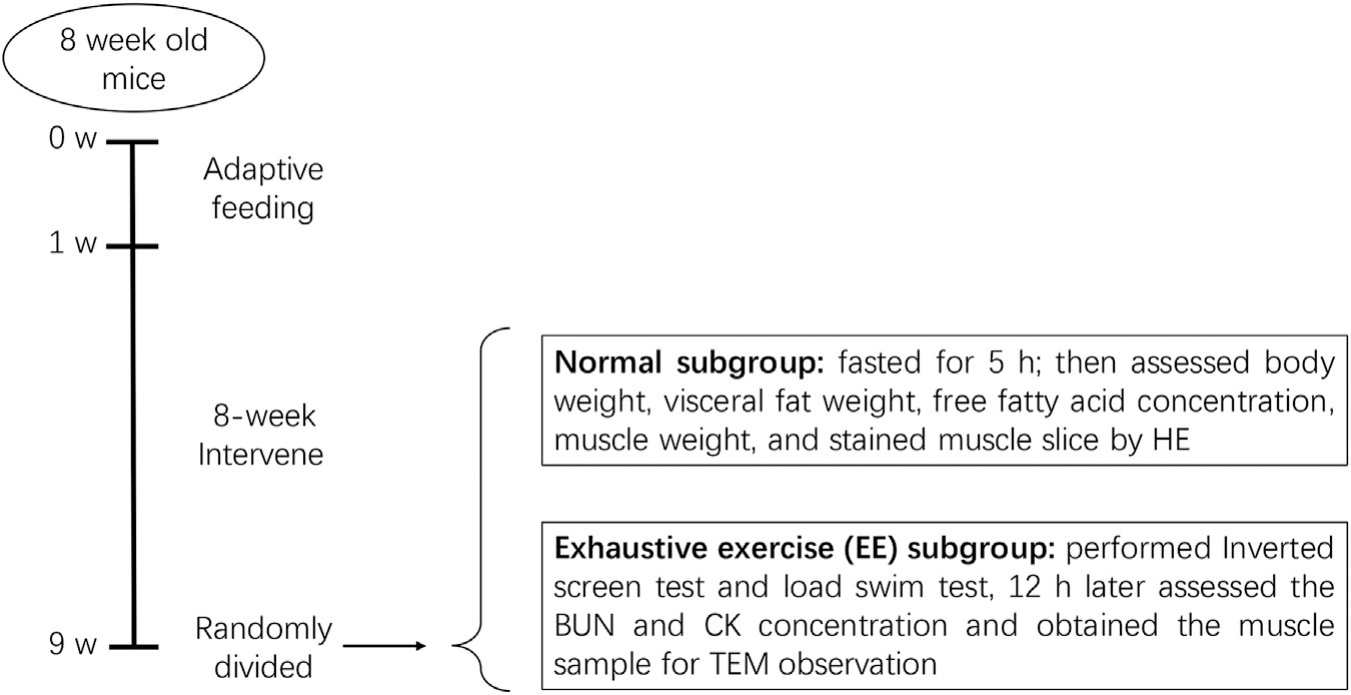
Diagram of animal experiments. After a 1 week adaptive feeding, mice were randomly divided into four groups: normal chow (NC), high-fat diet (HFD), HFD + *R. sacra* (HFD + RS) and HFD + exercise (HFD+EX). 8 weeks later, mice in each group were randomly divided into two subgroups: normal subgroup and exhaustive exercise (EE) subgroup, the subsequent experiments were performed as the diagram showed.

After 8 weeks, each group was further divided into two subgroups with or without exhaustive exercise (*n* = 8). The mice in the EE subgroups further underwent an inverted screen test and load swimming (5% body weight) session as previously described (Dun et al., 2017). After the experiments, all mice were anesthetized via an intraperitoneal injection of 1% pentobarbital sodium (150 mg/kg) and then sacrificed via exsanguination. Blood samples were collected from the inferior vena cava. Gastrocnemius muscle and visceral fat including mesenteric, epididymal, and perirenal fat tissue were dissected and weighted eventually. The protocol has been described in Figure 1.

### R. Sacra

The highly pure extract from the root of *Rhodiola chrysanthemifolia subsp. sacra (Raym.-Hamet) H.Ohba* (*R. sacra*) was provided by the Tibet Rhodiola Pharmaceutical Holding Company. Root of *R. sacra* was decocted with double distilled water twice, first time for 1.5 hours and second time for 1 hour, then collect the decoction after filtration. The filtrated liquor was concentrated to the relative density 1.25–1.30 (detected in 40°C) and prepared to powder by spray-drying. HPLC-MS analysis revealed that the main effective components are salidroside (C14H20O7, 2.62%) and flavone (C27H30O16, 3.27%) (Supplementary Figure S1 and Supplementary Table S1) and the exact preparation complied with The Chinese Pharmacopeia 2015 (inspection report number C1051612067). The extract powder was dissolved in distilled water (50 mg/ml) and it was administered to mice by gavage at a liquid/ body weight ratio of 0.1 ml/10 g for ten consecutive weeks (Every morning between 9 a.m. and 10 a. m.).

### Exercise Training

The mice in the HFD + EX group were subjected to a moderate intensity swim training as previously described (Dun et al., 2017). Briefly, the mice were placed in a Morris water maze pool (60 cm high and 120 cm in diameter) (XR-XM101-R, ZSdichuang, Beijing, China) for a swimming training from 10 to 60 min. Swimming exercise was performed between 9 a.m. to 2 p.m. and the mice exhibited minimal variations in aerobic capacity.

### Serum Free Fatty Acid

Mouse blood samples were collected and then assayed for serum free fatty acid according to the manufacturer’s instructions (A042, Nanjing Jiancheng Bioengineering Institute, China). Free fatty acid and coenzyme A (CoA) reaction and produce acetyl-CoA. Then, acetyl-CoA produce H2O2 with the acetyl CoA oxidase. We used peroxidase to make H_2_O_2_ colorized and quantify free fatty acid indirectly.

### Cross-Sectional Area of Muscle Fibers

Sliced muscle was stained by hematoxylin and eosin. Then, use the image analysis software Image J to measure area of each muscle fiber. Eventually, we statistically analyze the difference among groups.

### Inverted Screen Test

Mice were placed on the center of an invertible 40 × 40 cm wire screen with a padded surface. After gently inverting the screen, the time for hanging on and the limb strength was recorded.

### Exhaustive Exercise (EE) Program

The mice in EE subgroup (*n* = 8) performed a forced weight-loaded swimming session. The load composed of a lead sheath (0.8 mm thick, 0.5 cm wide) which is equal to 5% of each mouse body weight. The mice were enforced to swim till exhaustion, defined as the failure to rise the surface of water to breathe for 7 s. The swimming time was recorded and regarded as exercise capacity or muscular endurance.

### Transmission Electron Microscope (TEM)

The muscle tissues were resized to 1 × 1 × 3 mm^3^ and then fixed by 2.5% Glutaraldehyde and 1% osmic acid. After washed by 0.1 mol/L phosphate buffer, the tissue was dehydrated with acetone at a gradient concentration. Then embedding and solidify the tissue in 37°C for 12 hours and 60°C for 24 hours. After sliced to 50–100 nm and eventually they were examined by a transmission electron microscope (Tecnai G2 Spirit, FEI, United States) (Dun et al., 2017).

### Creatine Kinase (CK), and Blood Urea Nitrogen (BUN)

Blood samples were also collected from the EE subgroup 12 h after EE program. The CK concentration was determined via a colorimetric kit according to the manufacturer’s instructions (A032-1, Nanjing Jiancheng Bioengineering Institute, Nanjing, China). And the BUN was determined by a urease methods kit (C013-2, Nanjing Jiancheng Bioengineering Institute, Nanjing, China). The urea produced ammonia when exposed to urease, and ammonia could be colorized in the alkaline environment.

### Western Blot

Muscle tissues or cells were lysed in radioimmunoprecipitation assay (RIPA) buffer (Beyotime) containing 1 mmol/L phenylmethanesulfonyl fluoride (PMSF; Beyotime) on ice to extract proteins. After SDS-PAGE, the proteins were detected (Dun et al., 2017) and treated with primary antibodies against SIRT1, myosin heavy chain II (MyHC II), MuRF1, atrogin1, PGC1α, Microtubule-associated proteins 1A/1B light chain 3B (LC3), PTEN-induced kinase 1(PINK1), mitofusin-1 (MFN1), dynamin-related protein 1 (DRP1), citrate synthase (CS), and Glyceraldehyde 3-phosphate dehydrogenase (GAPDH) (Proteintech, Rosemont, IL, USA), respectively. Following HRP-labeled goat anti-rabbit IgG or goat anti-mice IgG (Proteintech), the bands were analyzed using a gel documentation system (Bio-Rad, Hercules, CA, United States).

### Adenosine Triphosphate (ATP) Content

The ATP content in muscle tissues and lysed cells were determined via phosphomolybdic acid colorimetric method according to the manufacturer’s instructions (A095-1-1, Nanjing Jiancheng Bioengineering Institute, Nanjing, China).

### Gene Expression Analysis

Total DNA was isolated from gastrocnemius using a DNeasy Kit (Qiagen). MtCO3 oligos and succinate dehydrogenase complex subunit A (SDHA) were analyzed to evaluate the quantification of mitochondrial and nuclear genomes. The primer sequences for the specific target genes are listed in the following Table 1.

**TABLE 1:** Primer sequences for qPCR analyses on tissues

Gene	Primer	Product length
M-DNA-mt-Co3	F GCA​GGA​TTC​TTC​TGA​GCG​TTC​T	67 bp
	R GTC​AGC​AGC​CTC​CTA​GAT​CAT​GT	
M-DNA-Sdha	F TACTACAGCCCCAAGTCT	194 bp
	R TGGACCCATCTTCTATGC	

### Cell Culture

C2C12 mouse myoblasts (Cobioer Biotechnology Ltd, Nanjing, China) were cultured in Dulbecco Modified Eagle Medium (DMEM) containing 10% fetal bovine serum and penicillin/streptomycin (5000U/5000 μg/ml; Gibco, Grand Island, NY, USA). Cells with a 75% confluence were incubated with differentiation media (DMEM containing 2% horse serum, Gibco) for 5 days. Our preliminary data suggested that the concentrations of salidroside (UPLC ≥98%, Sinopharm Chemical Reagent, Shanghai, China) above 50 μg/ml will induce cell death. Thus, our current study only used a concentration of salidroside less than 50 μg/ml to treat differentiated myotubes together with 0.75 mmol/L of palmitate for 24 hours, with or without pretreatment of 10 μmol/L EX-527 (SIRT1 inhibitor, 6-Chloro-2,3,4,9-tetrahydro-1H-Carbazole-1-carboxamide).

### SIRT1 Activity

Cells’ SIRT1 activity was measured by Colorimetric quantitative detection kit (Genmed Scientifics Inc. United States), each procedure follows the instruction.

### Immunofluorescence Staining

Cells were seeded on glass coverslips and incubated with anti-MyHC II (Proteintech). Following incubation with a fluorescent secondary antibody (Abcam, Cambridge, United Kingdom), images were acquired using a fluorescence microscope (Eclipse, Nikon, Japan).

### Oxygen Consumption Rate (OCR)

C2C12 myoblasts were seeded in XF 24-well microplates (Seahorse Bioscience, Billerica, MA, United States) and differentiated. Following treatments of mitochondrial inhibitors, including 1 μmol/L oligomycin, 1 μmol/L Carbonyl cyanide 4-(trifluoromethoxy) phenylhydrazone (FCCP) and 1 μmol/L rotenone/antimycin A, OCRs were measured with extracellular flux analysis (Seahorse Biosciences) every 8 minutes.

### Statistical Analysis

The results are expressed as Mean ± SEM. One-way ANOVA plus the Student-Newman-Keuls test was used for statistical analysis. *p* < 0.05 represents statistical significance.

## Results

### *R. sacra* Alleviates HFD-Induced Muscle Atrophy in Mice as Exercise

**FIGURE 2:**
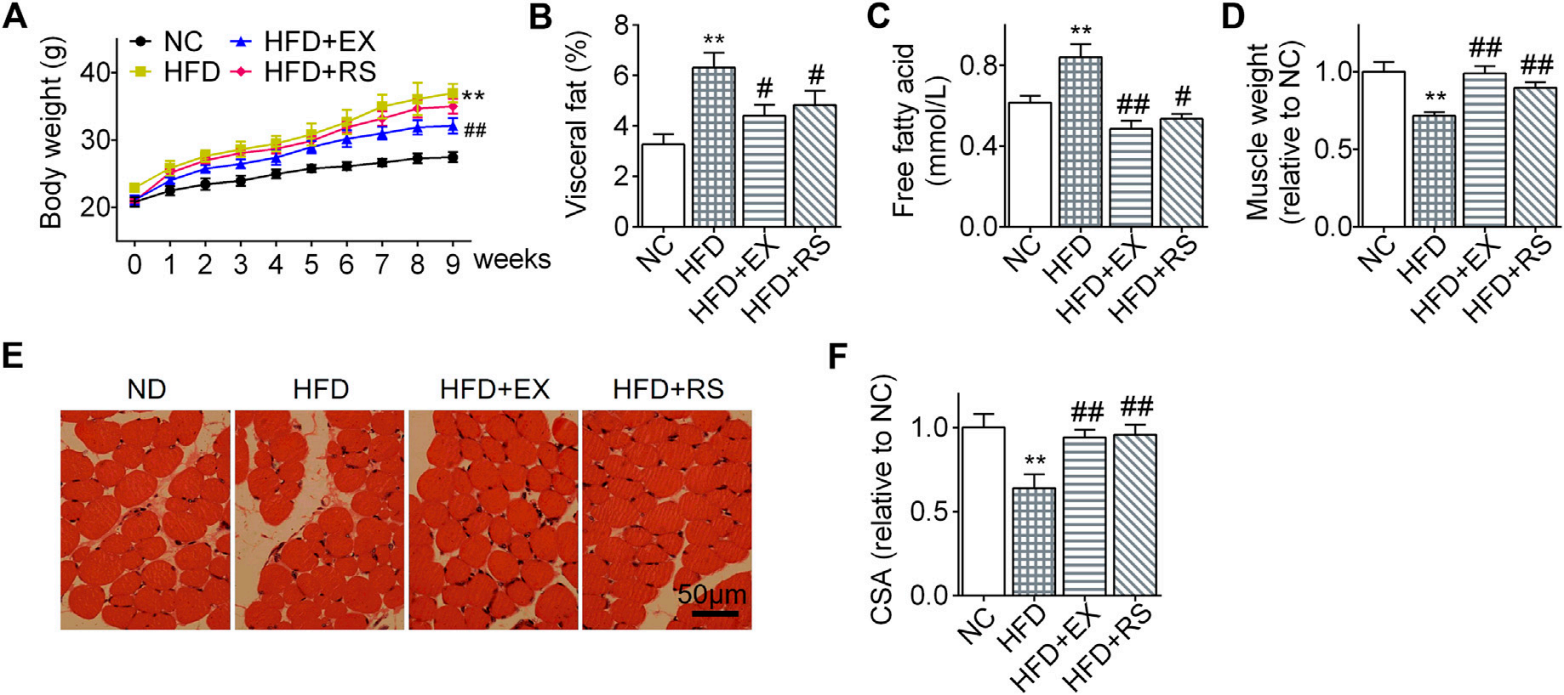
*R. sacra* alleviates HFD-induced muscle atrophy in mice as exercise **(A)** 8 week-old mice were fed normal chow (NC) or high-fat diet (HFD) with either *R. sacra* treatment (RS) or exercise training (EX) for 8 weeks, body weight was monitored in the whole process; **(B)** after 8 weeks, visceral fat/body weight, **(C)** serum free fatty acid, and **(D)** muscle weight/body weight were calculated and assessed; **(E)** Representative gastrocnemius muscle sections were stained with hematoxylin-eosin, **(F)** the cross-sectional area (CSA) of muscle fiber were measured. Scale bar = 50 μm. Data are expressed as Mean ± SEM, *n* = 8, ** represents *p* < 0.01 in comparison with NC; #, ## represent *p* < 0.05, *p* < 0.01 in comparison with HFD.

Lipid infiltration induced muscle atrophy in HFD mice (Tong et al., 2019). Exercise has been considered as an effective therapy to limit muscle atrophy or sarcopenia associated with disuse or denervation. However, it remains unclear how exercise affects HFD-induced muscle atrophy. In our present study, following HFD feeding for 8 weeks, mouse body weights increased more profound than normal chow group (Figure 2A). However, the body weight gain was significantly inhibited in the HFD group accompanied with exercise. Interestingly, HFD+RS mice showed a decreased tendency of body weight gain when compared to HFD group, but there is no statistical difference. The visceral fat weight (% body weigh) and serum free fatty acid in HFD mice were also increased following HFD. However, *R. sacra* or exercise decreased these two parameters (Figure 2B,1C). Gastrocnemius muscle weight was decreased following HFD feeding (Figure 2D) accompanied with a lower cross-sectional area of muscle fiber (Figure 2D). However, exercise training or *R. sacra* treatment significantly ameliorate these pathological conditions as showed in Figures 2D,E. These data collectively suggested that *R. sacra* treatment mimics exercise training to improve HFD-induced muscle atrophy.

### *R. sacra* or Exercise Improves Muscle Dysfunction in HFD Mice

**FIGURE 3:**
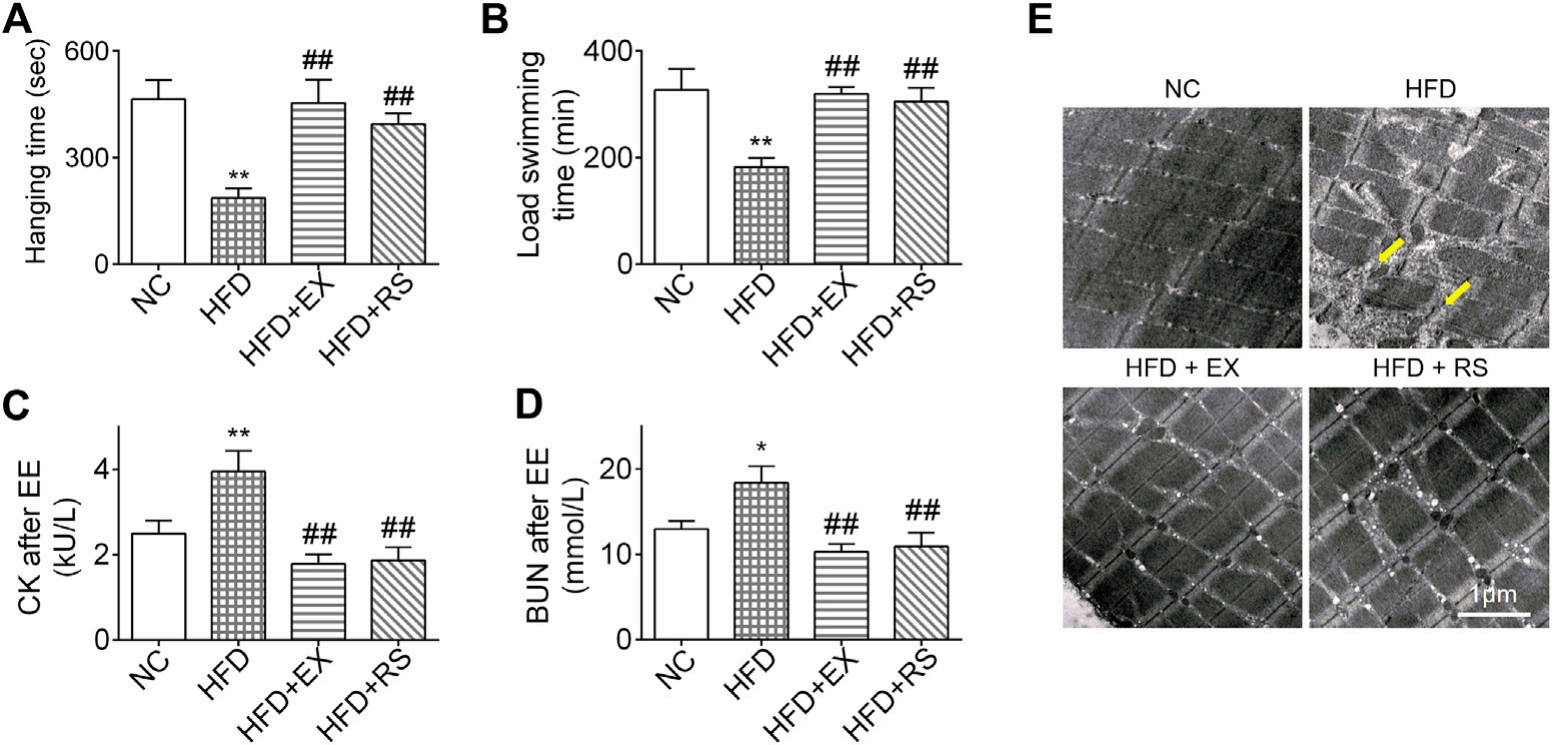
*R. sacra* or exercise improves muscle dysfunction in HFD mice **(A)** 8 week-old mice were fed normal chow (NC) or high-fat diet (HFD) with either *R. sacra* treatment (RS) or exercise training (EX) for 8 weeks, then the inverted screen test was performed and hanging time was analyzed; **(B)** and the forced weight-loaded swimming session were performed, and swimming time was analyzed; **(C)** 12 h after exhaustive exercise (EE), serum creatine kinase (CK), and **(D)** blood urea nitrogen (BUN) of mice were measured; **(E)** meanwhile, the morphology of muscle sample was observed by transmission electron microscope (TEM), and the damaged myofibrils (yellow arrows) were indicated in TEM images, scale bar = 1 μm. Data are expressed as Mean ± SEM, *n* = 3, *, ** represent *p* < 0.05, *p* < 0.01 in comparison with NC; #, ## represent *p* < 0.05, *p* < 0.01 in comparison with HFD.

We next performed inverted screen test and load swim test to assess muscle strength and endurance. The hanging time was decreased 50% in HFD group compared NC. However, accompanied with exercise or *R. sacra* treatment, the mice following HFD demonstrated a normal hanging time as NC group (Figure 3A). A similar result was also observed in the load swimming experiment (Figure 3B). Following load swimming, muscle damage was subsequently evaluated by electron microscopy. We found that in HFD group, muscle fiber consistency was disrupted, and mitochondrial membrane was ruptured (Figure 3E). In parallel, serum creatine kinase (CK) and BUN were also increased (Figures 3C,D). However, neither the morphologic changes in muscle cells nor CK and BUN release occur in HFD+EX or HFD+RS groups (Figures 3C–E), suggesting that exercise or *R. sacra* could improve muscle function deficiency induced by HFD.

### *R. sacra* Improves Atrophy Through Activating SIRT1 Signaling Pathway

**FIGURE 4:**
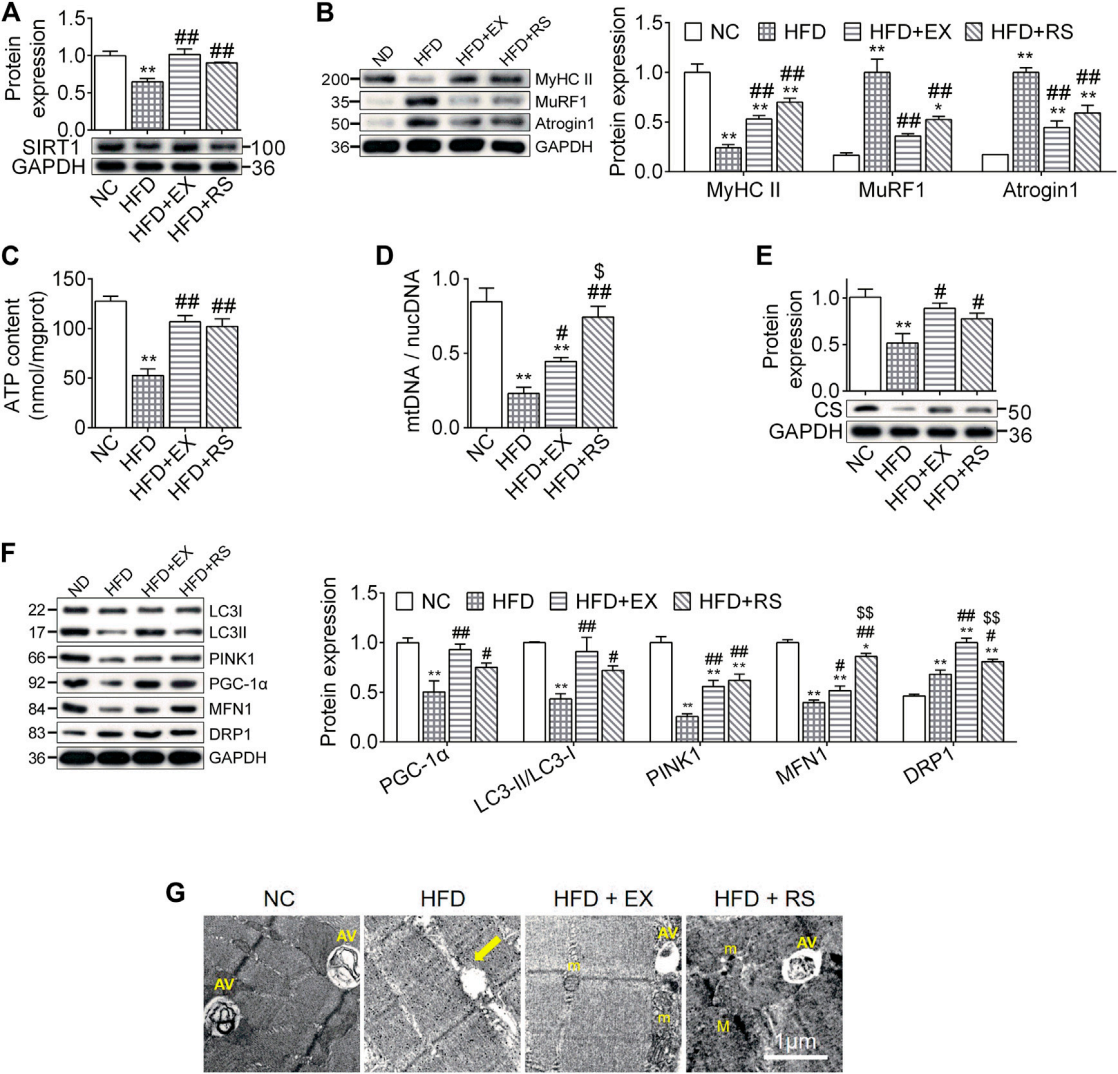
*R. sacra* improves atrophy through activating sirt1 signaling pathway **(A,B)** 8 week-old mice were fed normal chow (NC) or high-fat diet (HFD) with either *R. sacra* treatment (RS) or exercise training (EX) for 8 weeks, then muscle samples were obtained and the relative protein expression of SIRT1, MyHC II, MuRF1, and Atrogin1 were assessed; **(C)** the ATP content is measured; **(D)** the ratio between mitochondrial DNA (mtDNA) and nuclear DNA (nucDNA) determined; **(E,F)** the relative protein expression of CS, PGC-1α, LC3, PINK1, MFN1, and DRP1 were assessed; **(G)** the morphology of muscle sample was observed by transmission electron microscope (TEM), and the damaged mitochondrion (yellow arrows), normal mitochondria (m), autophagic vacuoles (AV), as well as myofilament (M) were indicated in TEM images, scale bar = 1 μm. Data are expressed as Mean ± SEM, *n* = 3, *, ** represent *p* < 0.05, *p* < 0.01 in comparison with NC; #, ## represent *p* < 0.05, *p* < 0.01 in comparison with HFD; $, $$ represent *p* < 0.05, *p* < 0.01 in comparison with HFD+EX.

Atrophy is associated with protein degeneration, inflammation, and mitochondrial dysfunction. SIRT1, a key regulator in nutrients/energy metabolism and cell fate signaling, also modulates atrophic genes and mitochondrial homeostasis (Tonkin et al., 2012). We found that SIRT1 protein levels were significantly decreased in skeletal muscle following HFD feeding, and this reduction was inhibited by exercise training or *R. sacra* treatment (Figure 4A). MyHC II content was lost almost 76% in muscles isolated from HFD group compared to NC (*p* < 0.01). The protein expression of atrogenes including MuRF1 and atrogin1 were increased in HFD group (Figure 4B). All these changes were partially corrected in HFD+EX and HFD+RS groups. Interestingly, exercise training seems to have greater effects than *R. sacra* treatment although there is no statistical significance.

ATP content is an important parameter to evaluate mitochondrial functions. In our study, we found that ATP content was significantly reduced in skeletal muscle isolated from HFD group. This reduction was accompanied with a decrease in the expression of mtDNA/nucDNA and CS (Figures 4C–E), suggesting that HFD might induce muscle mitochondrial dysfunction. Exercise or *R. sacra* facilitated mitochondrial function recovery by increasing ATP content, mtDNA/nucDNA and CS. HFD skeletal muscle also demonstrated a reduction in protein expression of mitochondrial biogenesis marker, PGC-1α, mitophagy markers, LC3-II and PINK1, and fusion marker MFN1. The fission marker DRP1 was increased. Exercise training or *R. sacra* treatment prevent these changes (Figure 4F). Eventually, by using TEM, we observed damaged mitochondria characterized with edema and reduction of cristae (yellow arrows) in HFD muscles but autophagic vacuoles (marked by AV) were hardly observed. Autophagic vacuoles were more abundant in NC, HFD+EX and HFD+RS.

### Salidroside Prevents Myotube Atrophy Through Activating SIRT1 Signaling

**FIGURE 5:**
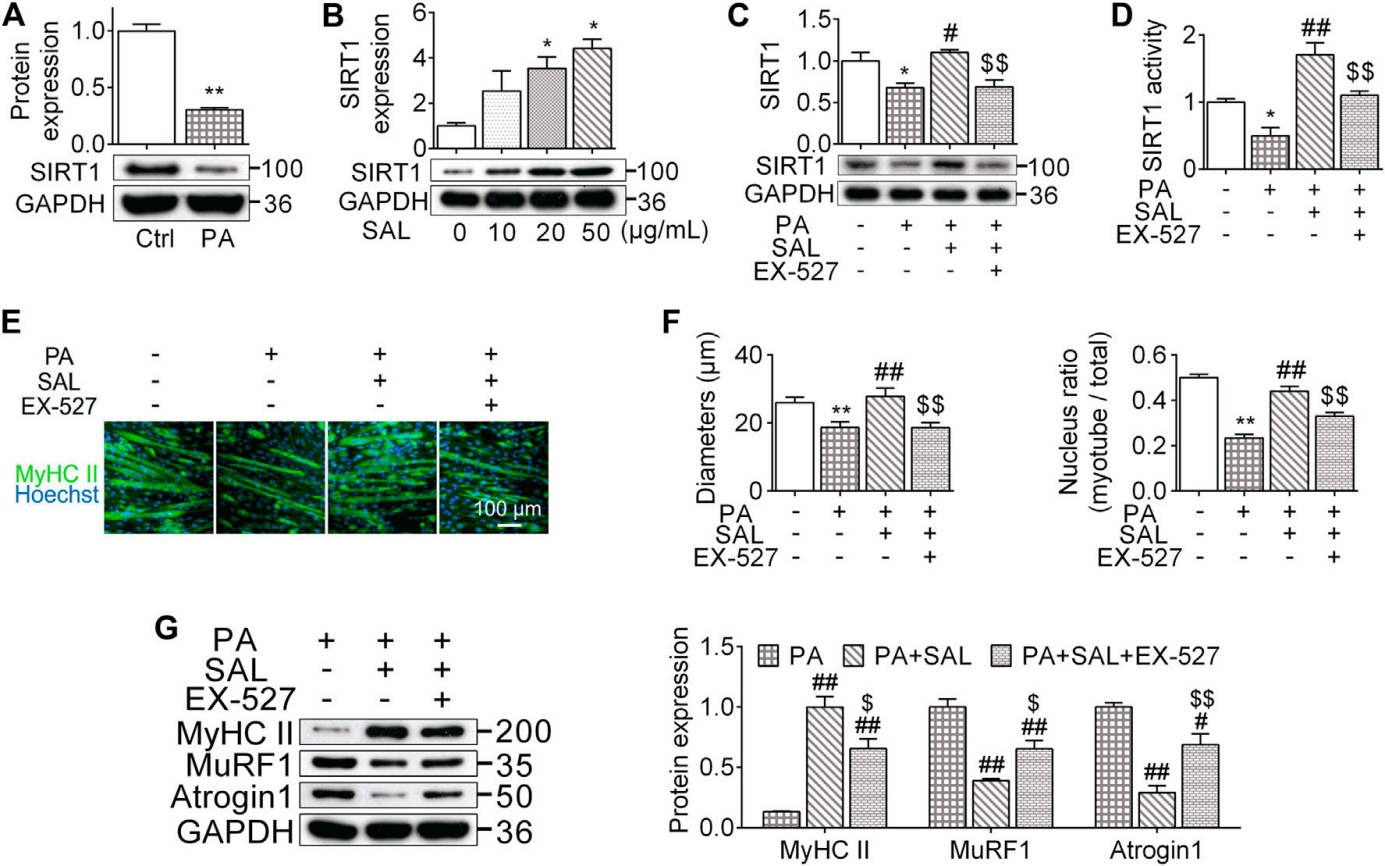
Salidroside prevents myotube atrophy through activating SIRT1 signaling. **(A)** The differentiated myotubes were vehicle (Ctrl) or 0.75 mmol/L palmitate (PA) for 24 hours, SIRT1 expression were subsequently measured; **(B)** the cells were incubated in different concentration of salidroside (SAL), then the protein expression of SIRT1 was measured; **(C,D)** the cells were treated with 50 μg/ml salidroside with or without 10 μmol/L EX-527 in the presence of 0.75mmol/L palmitate for 24 hours, then the protein expression and activity of SIRT1 were measured; **(E,F)** the morphology of differentiated myotubes was observe by MyHC II immunofluorescence, meanwhile the diameters and nucleus ratio of myotubes were calculated; **(G)** the relative protein expression of MyHC II, MuRF1, and Atrogin1 were assessed. Scale bar = 100 μm. Data are expressed as Mean ± SEM, *n* = 3, *, ** represent *p* < 0.05, *p* < 0.01 in comparison with Ctrl; #, ## represent *P* < 0.05, *P* < 0.01 in comparison with PA; $, $$ represent *p* < 0.05, *p* < 0.01 in comparison with PA+SAL.

To further investigate the molecular mechanisms by which *R. sacra* prevents HFD-induced muscle dysfunction, C2C12 myotubes were incubated with a high palmitate treatment. We found that high palmitate significantly inhibited the protein expression of SIRT1 in C2C12 myotubes as we observed *in vivo*, suggesting that palmitate might be a direct factor in accelerating muscle cell atrophy (Figure 5A). Salidroside, as the major active ingredient of *R. sacra,* activated SIRT1 in myotubes in a dose-dependent manner (Figure 5B). We therefore used salidroside to treat C2C12 myotubes with palmitate. We found that salidroside significantly relieve the inhibition of SIRT1 in palmitate-treated cells (Figures 5B–D).

We also analyzed the morphology of myotubes following 0.75 mM palmitate treatment. High palmitate significantly decreased the diameter of myotubes around 29% (*P* < 0.01) and reduced the number of nuclei located within the myosin heavy chain positive myotube by 44% (*p* < 0.01) (Figures 5E,4F). Salidroside abated these morphological alterations. Salidroside also upregulated MyHC II and decreased atrogenes expression (Figure 5G). To further clarify the role of SIRT1 signaling pathway in the influences of salidroside, a SIRT1 signaling inhibitor, EX-527, was used with salidroside in the presence of high palmitate in C2C12 myotubes. EX-527 prevented the effects of salidroside on upregulating SIRT1 expression and activity (Figures 5C,D). Meanwhile, the effects of salidroside on the myotube morphology, nucleus ratio, MyHC II content, as well as atrogenes were also abolished by EX-527 (Figures 5E–G). These data together suggest that salidroside limits myotube atrophy probably through activating the SIRT1 signaling pathway.

### Salidroside Activates Mitochondrial Quality Control Through the SIRT1 Signaling

**FIGURE 6:**
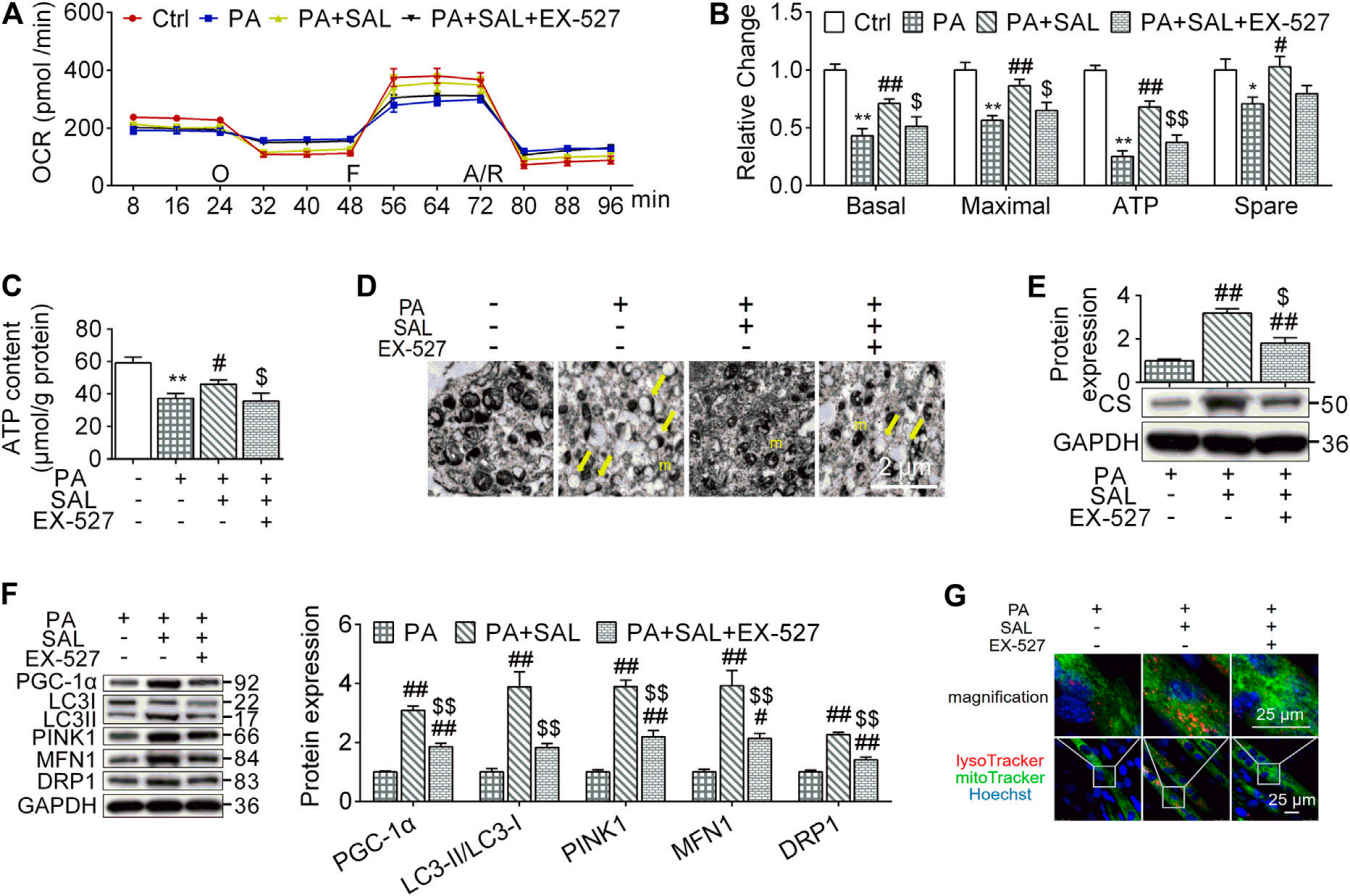
Salidroside activates mitochondrial quality control through the SIRT1 signaling. **(A,B)** the cells were treated with 50 μg/ml salidroside (SAL) with or without 10 μmol/L EX-527 in the presence of 0.75mmol/L palmitate (PA) for 24 hours, oxygen consumption rate (OCR) was measured by seahorse XF analyzer whereas 1 μmol/L oligomycin (O), 1 μmol/L FCCP (F) and 1 μmol/L rotenone/antimycin A (A/R) were added in order, then the basal respiration, maximal respiration, ATP respiration, and spare respiration were calculated from the OCR data; **(C)** ATP content were measured; **(D)** mitochondria in treated cells were directly observed by transmission electron microscope (TEM), and the normal mitochondria (m) as well as damaged mitochondria (yellow arrows) were indicated in TEM images, scale bar = 2 μm; **(D,F)** the protein expression of CS, PGC-1α, LC3, PINK1, MFN1, and DRP1 were measured; **(G)** the treated cells were stained by lysoTracker and mitoTracker, scale bar = 25 μm. Data are expressed as Mean ± SEM, *n* = 3, *, ** represent *p* < 0.05, *p* < 0.01 in comparison with Ctrl; #, ## represent *p* < 0.05, *p* < 0.01 in comparison with PA; $, $$ represent *p* < 0.05, *p* < 0.01 in comparison with PA+SAL.

Mitochondria maintain muscle contractile function and remodeling (Gan et al., 2018). In order to evaluate whether the beneficial effects of salidroside on skeletal muscle are associated with mitochondrial functions, we quantify mitochondrial functions and MQC-associated parameters. In this study, OCR levels, including basal respiration, maximal respiration, ATP production respiration, and spare respiration as well as ATP content were decreased in myotubes following palmitate-treatment. However, salidroside treatment prevented these reductions (Figures 6A–C). Similar results were observed in the expression of CS, the mitochondrial respiratory critical enzyme (Figure 6E). In morphology, we found that high palmitate induced a damage in mitochondrial structures characterized with edema and reduction of crista (yellow arrows). Salidroside treatment successfully mitigate the morphological changes in mitochondria (Figure 6D). However, EX-527 inhibited the improvement effects of salidroside on mitochondrial structure and functions evaluated by OCR levels, ATP content, CS expression, and TEM.

To explore how the activated SIRT1 signaling by salidroside regulates mitochondrial functions, we evaluated MQC. As shown in Figure 6F, salidroside treatment increased MQC quantified by PGC-1α, LC3-II, PINK1, MFN1, and DRP1 compared to PA group while EX-527 abolished this enhancement. Mitolysosome, a mitophagy marker, has been defined as the colocalization of LysoTraker and MitoTracker-stained organelles in cells. In the presence of SAL, Mitolysosomes were more abundant compared to palmitate-treated cells. However, addition of EX-527 reduced mitolysosomes content (Figure 6G). These results suggest that SIRT1 is a key regulator in modulating palmitate-induced MQC impairment and salidroside as an activator of SIRT1 might be a potential therapeutic treatment for HFD-induced muscle dysfunction.

## Discussion

HFD-induced muscle dysfunction is an important clinical issue due to its prevalence and poor prognosis. Exercise training improves muscle functions against atrophy (Perry et al., 2016). Our present study indicated that a long-term exercise mitigated HFD-induced muscle dysfunction via inhibiting atrogenes and enhancing MQC. Interestingly, *R. sacra* mimics long-term exercise training to mitigate muscle dysfunction and it also modulates SIRT1 signaling pathway, suggesting that *R. sacra* might be a new strategy to replace the long-term exercise training.

Diet containing 45% of fat (mainly lard) was used in this study to induce muscle dysfunction. It is long-chain saturated fatty acids (SFA) instead of unsaturated fatty acid described to be involved in lipotoxic pathways (Listenberger et al., 2003). Palmitate, as the most important SFA in lard (about 24%), was proposed to be an inducement of muscle mitochondrial dysfunction (Nisr et al., 2020). Thus, we used palmitate to establish dysfunctional C2C12 myotubes.

The NAD-dependent protein deacetylase, SIRT1, belongs to the sirtuin family and it performs a wide variety of functions in resisting metabolic disorders, cancer and cardiac stress etc.(Canto and Auwerx, 2012). Activation of SIRT1 by resveratrol also prevents muscle atrophy induced by mechanical unloading and dexamethasone (Lagouge et al., 2006; Momken et al., 2011). SIRT1 blocks FoxO1 and 3 and prevents the induction of key atrogenes atrogin1 and MuRF1 which are muscle-specific ubiquitin ligases contributing to proteolysis (Lee and Goldberg, 2013). Our present study found that SIRT1 activation also alleviates muscle dysfunction induced by HFD by reversing the upregulation of atrogenes in mice, suggesting that SIRT1 is an important signaling pathway in preventing or rescuing muscle dysfunction through the regulation of atrogenes.

Muscle dysfunction is associated with mitochondrial dysfunction (Gan et al., 2018), which is usually characterized with alteration of mitochondrial hemostasis, reduction of mitochondrial contents, and changed expression of mitochondrial genes which are responsible for oxidative metabolism in skeletal muscle (Joseph et al., 2012). Any cellular mechanism which improves mitochondrial functions would have important therapeutic potentials to resist the development of muscle dysfunction. SIRT1 activation protects mitochondrial oxidative functions. Supplementation of nicotinamide riboside, an NAD+ precursor, activates SIRT1 and upregulates mitochondrial energy metabolic genes in mice (Canto et al., 2012). Furthermore, an increase in mitochondrial respiration has been observed in permeabilized skeletal muscle fibers from human subjects following 2 weeks of supplementation with acipimox, another NAD+ precursor (van de Weijer et al., 2015). SIRT1 also improves obesity-associated metabolic diseases through deacetylating and activating mitochondrial biogenetic marker, PGC-1α (Lagouge et al., 2006). Thus, SIRT1-regulated mitochondrial biogenesis and oxidative metabolism may be another important mechanism to prevent or rescue muscle dysfunction.

Exercise counteracts deleterious muscle influence of aging and obesity via resisting lipid-induced protein degeneration or metabolic disorders in skeletal muscle (Heo et al., 2018). Thus, a 12-month exercise training increased thigh cross-sectional area and muscle mass in older-aged patient with diabetes mellitus (Mavros et al., 2014). Exercise also enhances mitochondrial oxidative functions via activating SIRT1 (Gurd, 2011). Indeed, our present study indicated that HFD decreased mitochondrial ATP content, DNA ratio, and CS expression in mouse muscle which are accompanied with attenuated mitochondrial functions. These effects were overcome by additional exercise training. MQC including mitochondrial biogenesis, autophagy, fusion, and fission is key to maintain mitochondrial homeostasis and oxidative function. MQC disorders result in mitochondrial dysfunction and pathological changes. Exercise enhances MQC and improves exercise capacity in healthy mice (Dun et al., 2017). Our present data suggest that MQC-related parameters, PGC-1α, LC3-II, PINK1, DRP1, and MFN1 were inhibited, whereas exercise relieve the MQC impairment, suggesting that exercise training mitigate HFD-induced muscle dysfunction probably through mediating atrogenes and MQC.

Extracts from the root of *Rhodiola* have been traditionally used to improve hypoxic tolerance for the people who ascend to high altitudes (Kelly, 2001). *Rhodiola* also improves exercise performance and anti-stress ability of skeletal muscle in human subjects and animal models which are probably associated with enhanced mitochondrial functions in skeletal muscle (Abidov et al., 2003; Noreen et al., 2013). We previously indicated that *R. sacra* triggered MQC and ultimately improves the exercise capacity and anti-fatigue ability in healthy mice (Dun et al., 2017). However, it remains uncertain whether *R. sacra* is beneficial to HFD-induced muscle dysfunction. In the present study, muscle atrophy and SIRT1 inhibition occurred in HFD-induced obese mice which were significantly mitigate by *R. sacra* treatment or exercise training. In parallel, HFD-impacted mitochondrial function and MQC in obese mice were also improved by *R. sacra* treatment or exercise training, suggesting that *R. sacra* has similar therapeutic effect as exercise training on activating SIRT1 and enhancing mitochondrial functions.

Salidroside is an active ingredient from the roots of *R. sacra*. It ameliorates muscle atrophy in cachexia or denervation via activating mTOR signaling pathway or reducing expression of atrogenes, such as MuRF1 and Atrogin-1 (Chen et al., 2016; Wu et al., 2019). Our previous study also indicated that salidroside resists metabolic disorders in skeletal muscle via upregulating SIRT1-mediated mitochondrial quality control in mice (You et al., 2020). Our present study further showed that in skeletal muscle cells, salidroside abated palmitate-induced SIRT1 inhibition, atrogenes activation, and myotubes atrophy and these therapeutic effects were significantly attenuated by additional EX-527 pre-treatment. These results indicate that salidroside may counteract lipid accumulation-caused atrophic factors and mitochondrial alteration by activating SIRT1 signal pathway. As the LC-MS analysis shown, polyphenols are main bioactive compounds of *R. sacra* too. Moreover, Capó and his colleagues found that polyphenolic extract ameliorate muscle decline in by reducing oxidative stress and oxidative damage (Annunziata et al., 2020). Thus, polyphenols might be responsible for the biological effects of *R. sacra* in the study and could be another potential treatment for HFD-induced muscle atrophy, but more data is needed.

**FIGURE 7:**
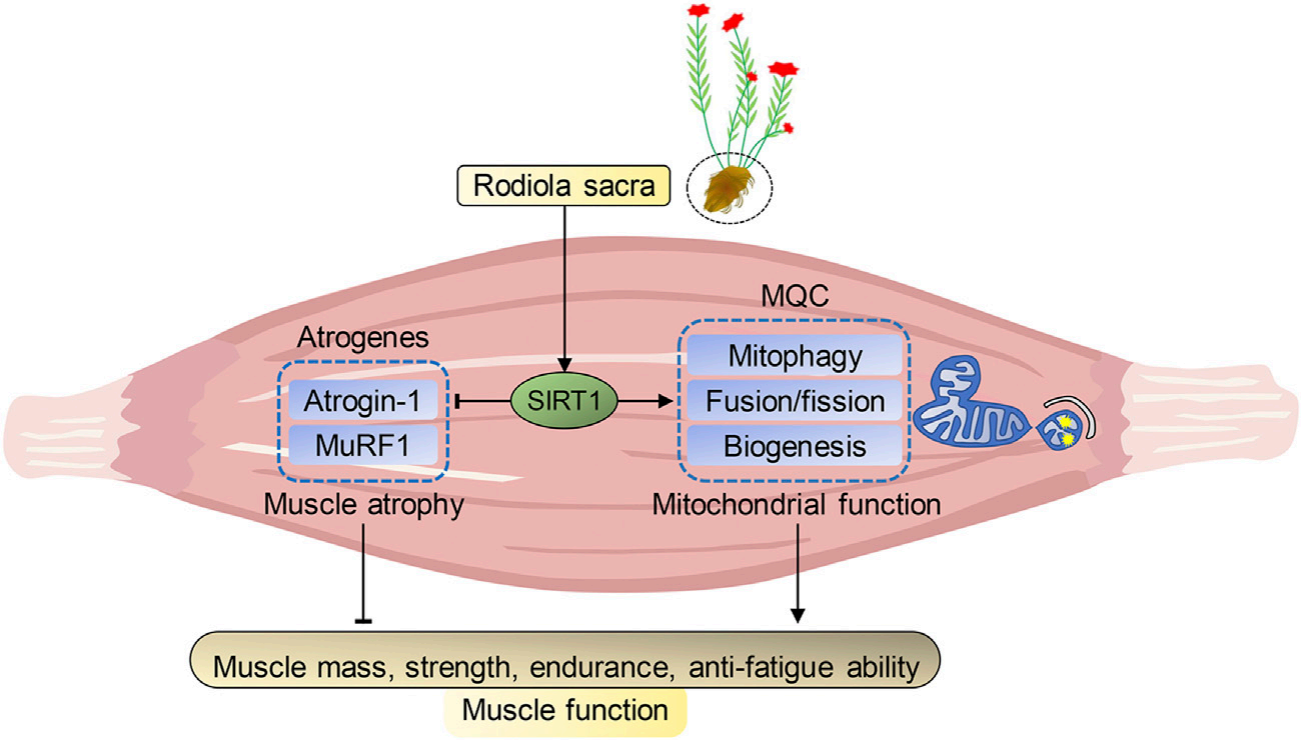
Proposed pathway of Rhodiola effects on HFD-induced muscle dysfunction. Sirtuin1 (SIRT1) is the key regulator of atrophy via mediating atrogenes and mitochondrial hemostasis. During high-fat diet (HFD)-induced obesity, SIRT1 in muscle was inhibited and adversely alteration in atrogenes and mitochondrial quality control (MQC) were observed. However, our data shows exercise training or *R. Sacra* treatment suppress atrogenes and enhances MQC to alleviate HFD-induced muscle dysfunction through activating SIRT1 signaling. Thus, we conclude that *R. sacra* treatment might be a potential exercise mimic or supplement therapy against HFD-induced muscle dysfunction.

In summary, our present study indicated that *R. sacra* mimics exercise to alleviate HFD-induced muscle dysfunction via inhibiting atrogenes and enhancing MQC via SIRT1 pathway, which was summarized in Figure 7. We believe that *R. sacra* treatment may be a potential substitute for long-term exercise training in clinical practice against muscle dysfunction in the future.

## Data Availability

The original contributions presented in the study are included in the article/Supplementary Material, further inquiries can be directed to the corresponding author.
