# Supplementary material for: The Treatment of Rhodiola Mimics Exercise to Resist High-Fat Diet-Induced Muscle Dysfunction via Sirtuin1-Dependent Mechanisms
Source: Front Pharmacol. 2021 Apr 15;12:646489. doi: 10.3389/fphar.2021.646489 (PMC8082455; doi:10.3389/fphar.2021.646489)
Supplement: Supplementary file 1 [file datasheet1.docx]

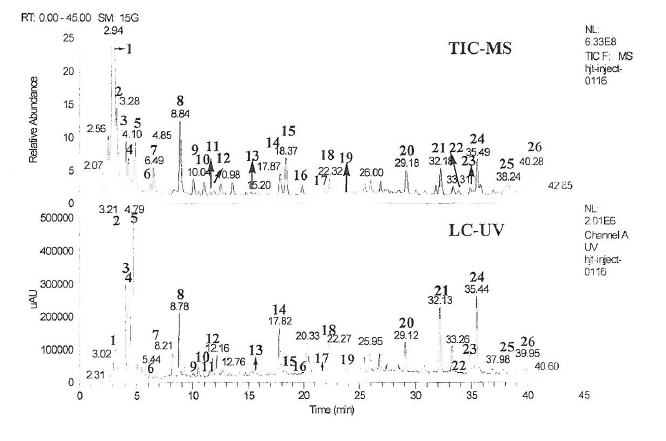


Figure S1. HPLC-MS analysis of R. sacra extract.

Table S1. LC-MS data and speculated structure of R. sacra components

| Peak  No. | t_R_  (min) | MS  (m/z) | Identity |
| --- | --- | --- | --- |
| 1 | 2.94 | 209, 269 |  |
| 2 | 3.28 | 191, 251 | 2,7-anhydro-β-D-heptul-opyranoses (192) |
| 3 | 4.10 | 320, 334 |  |
| 4 | 4.26 | 267, 347 |  |
| 5 | 4.85 | 359 |  |
| 6 | 6.22 | 304, 318, 356 |  |
| 7 | 8.80 | 299, 359 |  |
| 8 | 10.02 | 293, 359 | Salidroside (300) |
| 9 | 10.98 | 293, 307, 345 |  |
| 10 | 10.98 | 296, 358 |  |
| 11 | 11.52 | 289, 349 |  |
| 12 | 11.91 | 137 | Tyrosol (138) |
| 13 | 15.20 | 457, 517 |  |
| 14 | 17.87 | 451 | Parry glycosides (452) |
| 15 | 18.37 | 461, 475, 513 |  |
| 16 | 19.87 | 329, 343, 381 |  |
| 17 | 21.92 | 511, 563 |  |
| 18 | 22.32 | 609, 645, 669 | Rhodiosin (610) |
| 19 | 23.77 | 463, 561, 621 | Rhodionin (464) |
| 20 | 29.18 | 593, 653 |  |
| 21 | 32.18 | 785, 853 | 1,2,3,6-Tetragalyl -β-D-glucopyranose (786) |
| 22 | 33.82 | 939, 963 | 1,2,3,4,6-Pentagalloyl-β-D-glucopyranose (940) |
| 23 | 34.89 | 447, 507 | 3,5,7,8-tetrahydroxy- flavone-4’-oxygen-α-rhamnoside (488) |
| 24 | 35.80 | 445, 491, 505 | Geranyl 1-O-α-L-(1-6)-β-D-glucopyranoside (446) |
| 25 | 38.24 | 423, 467, 483 |  |
| 26 | 40.28 | 469, 483, 507 |  |
